# The cultural adaptation of the Friendship Bench Intervention to address perinatal psychological distress in Sierra Leone: an application of the ADAPT-ITT framework and the Ecological Validity Model

**DOI:** 10.3389/fpsyt.2025.1441936

**Published:** 2025-02-19

**Authors:** Abdulai Jawo Bah, Haja Ramatulai Wurie, Mohamed Samai, Rebecca Horn, Alastair Ager

**Affiliations:** ^1^ Institute for Global Health and Development, Queen Margaret University, Edinburgh, United Kingdom; ^2^ College of Medicine and Allied Health Sciences, University of SIerra Leone, Freetown, Sierra Leone

**Keywords:** cultural adaptation, perinatal, psychological distress, lay health worker, task-sharing

## Abstract

**Background:**

In Sierra Leone, women of reproductive age represent a significant portion of the population and face heightened mental health challenges due to the lasting effects of civil war, the Ebola epidemic, and the COVID-19 pandemic. This study aimed to culturally adapt the Friendship Bench Intervention (FBI) for perinatal psychological distress in Sierra Leone.

**Method:**

We utilized the ADAPT-ITT framework and Bernal’s Ecological Validity Model (EVM) for culturally adapting the FBI’s process and content. The adaptation stages included a formative study to assess perinatal women’s mental health needs. We screened the FBI for modifications based on the data from the formative study and EVM. The initial FBI manual was presented to mother-mother support groups (MMSGs, n=5) and primary health workers (n=3) for feedback (version 1.0). A theatre test with perinatal women (n=10) was conducted led by MMSGs, yielding further feedback (version 2.0). The revised manual was then reviewed by topical experts (n=2), whose insights were incorporated (version 3.0).

**Results:**

The Friendship Bench manual for Sierra Leone has been revised to better meet the cultural needs of perinatal women. The cover now illustrates an elderly woman conversing with a new mother, emphasizing community support. Culturally relevant idioms, such as “poil at” and “mind not steady,” replace previous terms, and new screening tools, the Sierra Leone Perinatal Psychological Distress Scale (SLPPDS) and the Function Scale, have been introduced. The problem-solving therapy was simplified from seven to four steps, and training duration was reduced from nine days to two, using visual aids to enhance comprehension for those with low literacy levels.

**Conclusion:**

Through this systematic approach, we successfully culturally adapted the FBI for treating perinatal psychological distress in Sierra Leone. The next step is to evaluate it feasibility, acceptability, and preliminary effectiveness in perinatal care settings.

## Introduction

The perinatal period if often associated with significant physiological, social, and psychological changes in women, often resulting in common perinatal mental disorders (CPMDs), with depression and anxiety being the most common (1). Global estimates indicate that the prevalence of CPMDs varies between 13% and 30%, with higher rates observed in low- and middle-income countries (LMICs; 2). In a recent study conducted in Kono district, in Eastern Sierra Leone, the prevalence of postnatal depression was 58.3% (Bah et al., in preparation). Despite the high prevalence of postnatal depression and the associated social and economic challenges among perinatal women due to the legacy of eleven years of civil war, the worst Ebola epidemic in human history, and the COVID-19 pandemic, evidence-based mental health interventions remain limited (3). In Sierra Leone, women of childbearing age (14-49) constitute about a quarter of the population (4). Some are often exposed to high rates of sex and gender-based violence, inter-partner violence, gender inequality, and lack of educational opportunities compared to men (5, 6), which is often associated with elevated rates of psychological distress (7). Furthermore, in Sierra Leone, women are disproportionately exposed to food insecurity, multi-dimensional poverty, and limited access to healthcare (7), which is compounded during the perinatal period. Women in rural areas reside in resource-limited settings plagued by high levels of gender inequality and patriarchy (5), exacerbating the negative impacts of previous trauma and loss. This situation contributes to increased rates of suicidal ideation, depression, hopelessness, stressful behaviours, low self-esteem, and increased maternal mortality among women (1, 6).

Enhancing maternal health is a fundamental priority of the Sustainable Development Goals and the Global Strategy for Women’s, Children’s, and Adolescents’ Health. However, much of the emphasis remains on physical health. While addressing physical health is essential—given that complications such as bleeding, infections, and eclampsia are leading causes of maternal mortality—neglecting mental health needs leaves mothers neglected (2). Sierra Leone has developed a comprehensive Reproductive Maternal Neonatal Child and Adolescent (RMNCH) policy (8). However, the absence of mental health considerations in the RMNCH program is concerning, especially given that perinatal mental health is in the National Mental Health Policy and Strategic Plan, first published in 2010 and updated in 2019 (9). Research indicates that neglecting perinatal common mental disorders can impact negatively on pregnancy outcomes, as seen in intra-uterine growth retardation, preterm delivery, and delayed social, physical, emotional and neuro-cognitive development (10). Studies have also established an association between maternal depression and decreased or lack of breastfeeding, infant malnutrition, increased absenteeism for immunization, and negative impact on physical and mental health throughout the life-course — with an intergenerational impact (11). Consequently, it is essential to increase government investments in evidence-based mental health and psychosocial support interventions, given that the evidence showed that the return on investment is 1:4 (12).

In recent years, the World Health Organization (WHO) has highlighted the critical need for scalable task-sharing psychological interventions in low-resource settings due to the significant gap between the demand for and availability of evidence-based mental health services in these settings (13). Low-intensity, manualized, task-sharing interventions focus on training non-specialist providers, such as community and lay health workers to deliver mental health care. Designing, implementing, monitoring and evaluating (DIME) new evidence-informed interventions can be resource-intensive; therefore, culturally adapting existing interventions may expedite their implementation especially in resource limited settings (14). Cultural adaptation is the systematic modification of the design and/or delivery of an evidence-based intervention to enhance its relevance and effectiveness in a specific setting different from where it was originally developed (15). Cultural beliefs significantly shape individuals’ understanding of mental health, often leading to stigma, decreased help-seeking, and often include family involvement in decision-making within collectivist societies (16). Additionally, therapeutic approaches may conflict with religious beliefs that challenge notions of personal agency. This adaptation process is essential for ensuring that the intervention is compatible with their values and beliefs, while also integrating their needs, priorities, and social context (17, 18). Meta-analyses have shown that culturally adapted interventions are generally more effective than non-adapted ones (19, 20). Additionally, cultural adaptation is an ethical responsibility, reducing the risk of imposing treatments that conflict with individual’s cultural norms (21). However, such modifications are frequently inadequately documented, which can hinder their replication and the systematic evaluation of their effectiveness (17). Therefore, employing a clear framework for adapting evidence-based interventions for new cultural contexts and populations—such as perinatal women—can enhance engagement, improve access to these services, and support robust methodological evaluations of their clinical effectiveness.

In Sierra Leone, previous initiatives have aimed to meet the mental health needs of conflict-affected youths. Many of these interventions adopt a transdiagnostic approach to address the co-occurrence of symptoms such as depression, anxiety, and post-traumatic stress (PTS), which are prevalent among children associated with armed forces and armed groups (CAAFAG) (22). For example, the Youth Readiness Intervention (YRI) has been effectively implemented for CAAFAG, resulting in significant reductions in post-traumatic stress symptoms (PTS), anxiety, depression, somatic complaints, and functional impairments while also improving quality of life (23). In a randomized controlled trial (RCT), the YRI showed that the intervention improved prosocial behaviours, emotion regulation, decreased school dropouts, improved classroom behaviour and social support (24). Despite the positive outcomes associated with the YRI, there remains a pressing need for interventions specifically tailored to the unique needs of pregnant women and new mothers in Sierra Leone.

## The current study

This research aimed to identify and culturally adapt a scalable, evidence-based mental health intervention to address perinatal psychological distress in Sierra Leone. We chose the FBI (see **Table 1**) for this study. The Friendship Bench intervention originally developed in Zimbabwe, is a community-based mental health initiative designed to provide accessible psychological support through trained lay health workers. It employs a simple, culturally appropriate model where individuals receive counseling on a bench in their community, facilitating open dialogue about mental health issues using a problem-solving approach (25). This program has shown significant effectiveness in reducing symptoms of depression and anxiety among participants, demonstrating the potential as a scalable, low-cost, mental health solution (26). By integrating mental health care into community settings, the FBI serves as a proof of concept for similar interventions globally.

**Table 1:** Description of the original Friendship Bench Intervention.

Theoretical	Based on problem-solving therapy
delivery agent	Lay health workers (health promoters)
Structure of intervention	Six weekly sessions of 30–45 min delivered through the Friendship Bench over 6 weeks, including home visits were deemed necessary. The first session lasting between 45 and 60 min
Structure and sessions and areas covered	Part 1. Problem identification (*Kuvhura pfungwa*): (A) Share Shona Symptom Questionnaire (SSQ) information with client, explain symptoms in relation to kufungisisa, (B) Actively listen to clients story, identify and list problems raised, clearly define problem/s. Problem exploration (*kusimudzira*): (C) understand the story, help client prioritise problems by summarizing and asking if you have missed anything, (D) brainstorm practical/feasible solutions, outline the options available (these have to come from client), encourage client to think over solutions of each problem before having the client decide which one to focus on. Help client to come up with a specific, measurable, achievable and realistic solution (don’t tell client what to do). Agree what the client will do before you next meet and set appointment date Part 2. Reassure (*Kusimbisa*) (E) Home visit if needed before second meeting, otherwise see again on the bench, how did it go? Went well, then reassure praise encourage. If no progress or new obstacles present then go back to Part 1 contents, redefine problem and goals, what were the obstacles? Problem solves around obstacles and give homework again and reassure, you can phone or send SMS to reassure client (up to 6 per client). Part 3 (kusimbisisa). (F) Summarise session 1, how did it go? Going well then reassure and encourage. Still having problems with agreed plan? Go back to Part 1 again or if you feel frustrated go to supervisor
Remember	Action plan: (G) Zero in on a specific solution, focus on what client wants to do and not what you think should be done, (H) How, when, what assistance is needed? Referral if necessary (I). Identify activities the person used to find rewarding and which matter to them and encourage these (J), Implementation: (K) How will it be done? Motivate; homework, refer after 4th session to support group. Follow up: (L) What has been achieved? What were/are the obstacles if any? Go back to Part 1 as often as needed during the 6 sessions (M) Reinforce. What has been achieved, repeat SSQ score. (N) No improvement refers to supervisor. Nurse counsellor
Tools	SSQ-14, Friendship bench manual, referral protocol
Training	Two weeks training before onset of FB, ongoing training every two weeks for the first six months, thereafter monthly
Supervision	Weekly group supervision by a clinician (Psychologist) or senior study team member trained in PST. Access to direct mobile voice call to support team

The cultural adaptation process followed a structured approach based on the ADAPT-ITT framework (18). This framework has been widely applied for cultural adaptations in health and mental health research across various LMICs and among marginalized groups in Vietnam (27), Columbia (28), Sierra Leone (29), and Ethiopia (30). Furthermore, we combined it with the Ecological Validity Model (EVM) to inform the content modifications of the FBI manual (31), which systematizes adaptations according to eight key parameters: language, goal, context, concept, content, methodology, person, and metaphor. Combining ADAPT ITT and EVM (30) aligns with the call for the use of multiple adaptation models (32), which is considered a strength in the cultural adaptation of evidence-based interventions developed in the Global North for culturally diverse populations in resource-limited settings in the Global South.

## Method

### Study design

This qualitative research is nested in an exploratory, sequential mixed-method study conducted between January 2020 and July 2021. This study was conducted in Krio, the primary lingua franca of Sierra Leone, which serves as the common language for communication among various ethnic groups, despite English being the official language (33).

### Study settings

This research was conducted at two communities, Campbell Town and Lumpa, randomly selected among a list of communities in Waterloo, part of the Freetown metropolitan area. Waterloo, a peri-urban area, is located in the Western Area Rural district, which is one of 16 districts in Sierra Leone. It is situated 20 miles from the capital city of Freetown. It is the second largest city in the Western Area after Freetown. It has a population of 55000 (4). Waterloo is one of the most ethnically diverse, peri-urban cities, and it lies on the main highway linking the capital city and rest of the country.

We worked with nurses, midwives, community health officers (CHOs), community members, pregnant women, new mothers, and MMGs in these communities for the cultural adaptation of the intervention. The primary language used in these communities is Krio, and their main economic activity is agriculture. Waterloo has one primary hospital as well as eight health centres that serve approximately 5,000 households each. Additionally, each health centre is connected to 3-5 health posts, which are basic healthcare facilities that conduct health promotion and illness prevention activities. CHOs head each community health centre (CHC), and their responsibilities include maintaining a list of perinatal women in their catchment area. Following the launch of the National Mental Health Policy and Strategy of Sierra Leone (9), some of the CHOs at these facilities were trained using the WHO mhGAP intervention guide, and are supported in providing first-line mental healthcare.

### Research team

The cultural adaptation was done by the study lead (AJB) and four research assistants, all University graduates who were part of the formative study, and understood the purpose of the intervention. A one-day training was conducted for the research assistants on the cultural adaptation of evidence-based interventions. The training was focused on ethics and techniques in cultural adaptation using the qualitative data we collected. All research assistants were involved in the project from the inception phase (34). One of the research assistants was a nurse, and previously worked as the mental health and psychosocial support lead at the Sierra Leone National Ambulance Services. We had two graphic designers in the education department of the Ministry of Health and Sanitation. A supervisory team at Queen Margaret University (AA & RH) supported us throughout the process.

### Cultural adaptation of the friendship bench intervention

There are eight sequential Phases in the ADAPT-ITT model (see **Table 2**): Phase 1, the assessment, was the formative study that assessed the mental health needs of pregnant women and new mothers using a rapid ethnographic approach (34). Phase 2, the decision was reached by reviewing the qualitative study and literature data. This paper describes the third to the sixth phases of the model—phase 3 (adaptation), phase 4 (production), phase 5 (topical experts), and phase 6 (integration). Phase 7 (training) and phase 8 (testing) will be published in a future study exploring the intervention’s feasibility, acceptability and effectiveness.

**Table 2:** The ADAPT—ITT framework.

No.	Phase	Method	Version
1	Assessment of study population	- Conduct free listing, pile sorting and key informant interviews with pregnant women, new mothers and men, and. A needs assessment - Analyse results of formative study	Completed in 2020
2	Decision on choosing Evidence based intervention for adaptation	- Decide which intervention to adapt based on literature review and similarity of the context - Decide on initial adaptations based on the needs assessment findings - Produce first draft of the culturally adapted manual for the intervention	Completed 2021 Version 1.0
3	Adaptation (using Bernal’s framework)	- Use the theatre test of intervention with MMSGs and perinatal women - Review the test feedback	Version 2.0 & Version 3.0
4	Production	- Produce a third draft of the culturally adapted manual	Version 3.0
5	Topical Experts	- Engage topical experts (supervisors; expert in cross-cultural study) - Review session content and provide inputs	Version 4.0
6	Integration	- Integrate the feedback from the topical experts into the manual	Version 4.0
7	Training	- Train the MMSGs piloting the culturally adapted FBI - Obtain feedback from the MMSGs piloting the intervention	
8	Testing	- Pilot test the culturally adapted manual for its feasibility, acceptability, and preliminary effectiveness. - Make any further revision and inputs to the tailored manual - Administer pre- and post-intervention assessments	

## Phase 1: assessment

This was a formative study, rapid ethnographic research nested in a larger study, which was a mixed-method, exploratory, sequential design. This Phase involved assessing the mental health needs of pregnant women and new mothers, including how they experience and express psychological distress, their coping mechanisms and help-seeking behaviour. We recruited pregnant women, new mothers, men, and older women at the community level in four districts (Bo, Western Area, Bombali and Kono) that is representative of the four geopolitical regions of the country. We conducted free listing (n=96), pile sorting (n=8) and key informant interviews (n=16), exploring the problems experienced by perinatal women related to thoughts, feelings and behaviours. This qualitative formative research highlighted a significant link between the depressive symptoms experienced by perinatal women and various stressors, including poverty, unemployment, insufficient partner support, abuse, bereavement, and unplanned or unwanted pregnancies (34). We analyzed the data using thematic content analysis, supported by frequency analysis and multidimensional scaling.

The analysis identified twenty signs of distress, and the concept of the self for perinatal women encompassed the heart, mind, and body, reflecting their emotional states such as sadness, stress, loneliness, and anger. They articulated various culturally specific idioms of distress, such as “stres” (stress) and “poil at” (depression), which are linked to broader issues like poverty, marital conflict, and gender inequality. These idioms function as overlapping indicators of distress, which can intensify over time. Commonly cited sources of psychological distress included interpersonal conflicts, economic hardship, and gender-related challenges (35). Notably, women did not perceive their problems as biomedical in nature, which is reflected in their coping strategies and help-seeking behaviours. Their coping mechanisms involved conversations with family, friends, community members, and engaging in risky behaviour such as drinking alcohol (34). However, many participants felt their strategies were ineffective, as unresolved issues persisted, contributing to ongoing stress. Help-seeking behaviours included reaching out to social networks, religious leaders, and occasionally traditional healers, while mother-to-mother support groups (MMSGs) were frequently mentioned as a local source of emotional support. The MMSGs in Sierra Leone are lay women, volunteers, working with the directorate of nutrition, supported by UNICEF. They empower perinatal women through peer support and education on health, nutrition, and hygiene to improve maternal and child health outcomes in Sierra Leone.

## Phase 2: decision

This Phase entailed reviewing the existing evidence-based interventions in the literature and deciding which one resonates with the findings from the formative study. To evaluate various interventions addressing perinatal psychological distress, we conducted a systematic review. The review involved a comprehensive search of the literature and existing programs, focusing on their effectiveness, cultural relevance, feasibility, and potential for adaptation (see **Table 3**). The review showed that culturally adapted interventions conducted by lay health workers in sub-Saharan African countries fell into three categories: IPT (41–45), FBI-PST (25, 36, 37, 46) and CBT (23, 24, 38–40). We selected the FBI due to its strong cultural fit within the collectivist context of Sierra Leone, where community relationships are vital, and the attributes and risk profiles of the perinatal women match with problem-solving theory and design approaches to interventions.

**Table 3:** Comparison of lay health worker delivered psychological interventions in LMICs.

Intervention	Description	Effectiveness	Cultural relevance	Feasibility	Adaptation potential
Friendship Bench (problem solving therapy) Chibanda et al., 2011 (25) Haas et al., 2023 (36) Chibanda et al., 2016 (37)	A community-based talk therapy intervention using lay counselors.	Effective in reducing depression and anxiety	Strong community ties; fits within local support structures.	High; utilizes existing community resources.	High; adaptable to local beliefs and practices.
Cognitive Behavioural Therapy (CBT) Rahman et al., 2008 (38) Murray et al., 2013 (39) Etrl et al., 2011 (40)	Structured psychotherapy focusing on changing negative thought patterns.	Evidence-based, but less effective in low-resource settings without trained therapists	May not align with local beliefs about mental health.	Moderate; requires trained professionals.	Limited; rigid structure may not resonate culturally.
Group Inter-Personal Therapy Bolton., 2003 (41) Bass., 2006 (42) Bolton et al., 2007 (43) Petersen et al., 2012 (44) Petersen at al., 2014 (45)	A structured group therapy format that encourages sharing and interpersonal connection.	Effective in enhancing social support and reducing isolation.	Highly relevant; fosters community connection and understanding.	Moderate; can be facilitated by trained peers. Shame can be a challenge for group sessions in the initial phase	High; easily adaptable to different cultural norms.

The formative study conducted in Sierra Leone presents compelling reasons for choosing the Friendship Bench intervention over Cognitive Behavioral Therapy (CBT) and Interpersonal Therapy (IPT) to address the mental health needs of perinatal women. Firstly, the study underscores the importance of cultural context, revealing that perinatal women express psychological distress through culturally specific idioms, which the Friendship Bench is well-equipped to address, unlike CBT and IPT that may lack cultural resonance (34). Additionally, the findings highlight the significance of community engagement, as participants favored seeking support from family and mother-to-mother support groups (MMSGs), which the Friendship Bench effectively taps into by employing trained lay counselors from the community (35).

Furthermore, the study indicates that participants view their psychological issues as linked to socio-economic stressors rather than biomedical concerns, aligning more closely with the Friendship Bench’s focus on social support and problem-solving (34). The intervention also leverages existing support structures like mother-to-mother support groups, enhancing its effectiveness without requiring extensive infrastructure or training, which is often a barrier for CBT and IPT in resource-limited settings. Moreover, the Friendship Bench emphasizes practical coping strategies and peer support, addressing the immediate needs expressed by participants for more effective coping mechanisms (34). Finally, while CBT and IPT are established therapies, the Friendship Bench has demonstrated effectiveness in similar low-resource contexts, suggesting it can lead to significant improvements in mental health outcomes among populations facing socio-economic challenges (25, 36, 37, see **Table 4**).

**Table 4:** Scientific evidence of the FBI in Zimbabwe.

Study	Aim	Sample and design	Results
FBI pilot trial (36)	To gather preliminary data on the effectiveness of a lay health worker delivered intervention and to see if the intervention would be feasible, and preliminary effectiveness	Non-randomized intervention 320 people were recruited, and attended six sessions of PST delivered by 10 lay health workers trained for 8 days.	There is preliminary evidence that lay primary health care workers can deliver locally adapted problem- solving therapy in Harare, Zimbabwe and that this can be associated with a meaningful reduction in symptoms of depression and common mental disorders. 3 and 6 sessions the mean score of the SSQ (Shona Symptom Questionnaire) dropped by 4.8 points to 6.5 (s.d = 2.4) [t = 13.6 (p = 0.0087)]
FBI Randomized Control Trial (25)	To evaluate the effectiveness of a culturally adapted psychological intervention for common mental disorders delivered by lay health worker in primary care.	Randomized clinical trials. 573 randomized patients (286 in the intervention group and 287 in the control group). 521 completed follow-ups at 6 months	Intervention group participants had fewer symptoms than control group participants on Shona Symptoms Questionnaire. The intervention group participants also had lower risk of symptoms of depression (13.7%; ARR, 0.28; 95% CI 0.22 to 0.34; p < 0.001)
FBI Randomized Controlled Trial (37)	To assess the effect of a lay health worker–led psychological intervention on ART adherence, virologic suppression, and mental health symptoms.	Cluster randomized design. A total of 516 participants (≥ 18 years old), were recruited (244 in Friendship Bench and 272 in enhanced standard care). Data were collected at 3,6,9 and 12 months using PHQ-9 and Shona checklist-14	The intervention had no statistically significant effect on adherence or viral suppression. There was an improvement in common mental disorders symptoms. The declines in SSQ-14 and PHQ-9 scores from baseline to 3 months 6 months and 9 months (were greater in the Friendship Bench than the standard care group.

Culturally adapting the Friendship Bench intervention from Zimbabwe to Sierra Leone requires consideration of several key differences. In Zimbabwe, due to the high literacy level, community engagement and awareness about mental health are more pronounced, potentially reducing stigma compared to Sierra Leone, where such awareness may be limited. Language diversity in Sierra Leone includes Krio and various local dialects, necessitating it translation and culturally relevant metaphors. The context of Sierra Leone involves distinct social norms and communal values that may differ from Zimbabwean practices. While Zimbabwe employs trained community health workers, Sierra Leone can leverage on mother-to-mother support groups (MMSGs) for delivery, enhancing relatability and trust.

## Phase 3: adaptation of the content using the EVM

The first author (AJB) and the research assistants read the FBI intervention manual to identify components of the intervention that could be subjected to cultural adaptation (adaptive hypothesis) across the eight EVM dimensions. Using the data from the formative study and the EVM, the original FBI manual was modified accordingly by the research team (version 1.0).

### Step 1. Presentation of the FBI to the MMSGs, healthcare workers and perinatal women

The research lead (AJB) and the nurse research assistant with a background in mental health psychosocial support presented the modified Zimbabwean FBI manual (version 1.0) as a training course to the MMSGs (n=5), and the primary healthcare workers, nurse (n=1), midwife (n=1), and a CHO (n=1), from the CHC for a two-day session. The other two research assistants served as note-takers. Following the presentation, we conducted a full-day adaptation workshop with the attendees looking at potential challenges and opportunities. MMSGs participated in and led several theatre testing simulations of a lay-health worker-delivered model. They role-played, and after each role-play, we discussed the modified delivery method and adaptations needed (18). MMSGs shared their concerns and thoughts about the barriers and facilitators that may impact the delivery model and potential implementation strategies to address the obstacles. We discussed the feedback from this workshop using the framework from the EVM, and the output from this workshop was the second version of the intervention manual (version 2.0).

### Step 2. Theatre testing

Theatre testing is a systematic approach for culturally adapting an intervention that forms part of the ADAPT-ITT approach (18). MMSGs presented version 2.0 of the adapted intervention with separate sessions for pregnant women (n=5) and new mothers (n=5) using theatre testing in the study site area. Clinical vignettes developed from the qualitative study were used for the demonstration. A MMSG (taking the therapist’s role), a pregnant woman, and later a new mother (playing the perinatal woman’s role) role-played the intervention sessions. Role-play activities simulated what the MMSGs could experience when delivering the culturally adapted intervention and what the perinatal women might experience when receiving it.

At the end of each demonstrated session, two facilitators from the research team led separate group discussions using the EVM (47) to improve the comprehension, relevance, and acceptability of the content. The note-takers captured the discussions and feedback. AJB and one of the research assistants analyzed these notes using thematic content analysis, using the EVM domains (version 3.0).

## Phase 4: production

In this Phase, the research team combined and analyzed all the data collected during the adaptation phases including the theatre testing, to produce the version 3.0 of the culturally adapted FBI.

## Phase 5 and 6: topical expert and integration

Version 3.0 of the manual was then reviewed by the PhD supervisory team at the Queen Margaret University (AA & RH) serving in the capacity of topical experts. Their suggestions and recommendations were integrated, leading to version 4.0 of the intervention manual. The two experts had significant expertise in cross-cultural mental health research, with experience working in low-resource settings, including Sierra Leone (7, 35). Following their inputs, the research team convened to evaluate the suggestions and determine the modifications to be made based on the cultural context. The experts’ feedback emphasized the cultural relevance, format, and duration of the intervention while ensuring the retention of the evidence-based component.

### Ethical approval

This study received ethical approval from the Institutional Review Board at Queen Margaret University and the Sierra Leone Ethics and Scientific Review Committee.

## Results

In this section, we present the findings from our study on the cultural adaptation of the FBI using the ADAPT ITT framework and the EVM. The ADAPT ITT framework comprehensively outlines the process for adapting the culturally modified FBI manual, detailing how each step maintains fidelity to the original intervention while addressing the unique needs of perinatal women. Following this, the EVM provides insights into the cultural adaptation of the manual, highlighting the essential adjustments made to align the content, context and delivery of the culturally adapted FBI with the cultural context of the intended perinatal women.

### ADAPT ITT

#### Phase 3: adaptation – the EVM (used as a framework for the content adaptation)

This section synthesizes findings across eight domains of the ecological validity model, highlighting key themes and participant quotes that illustrate the effectiveness of the intervention designed for perinatal mental health in Sierra Leone.

### The ecological validity model

The EVM outlines eight domains essential for culturally adapting evidence-based interventions: language, goals, methods, metaphor, content, context, concepts, and persons. Each domain is documented in a matrix format to detail the adaptation process (**Additional File IV** in **Supplementary Material**). For instance, training materials for MMSGs were initially developed in English and translated into Krio, ensuring clarity and cultural relevance. Language specificity was emphasized, using local idioms to enhance understanding and reduce stigma. Frequently used local idioms of psychological distress from the formative study: “*vex, heng at, stres, tink too much, cry cry, dikoraj*, and *fostrate”* were identified. All technical terms were translated into local expressions, and we replaced the term depression for example with culturally congruent terms such as “*poil at”*,” *at nor swit”*, and anxiety, with ‘‘*stres”* or *wori”*, and we tried to avoid psychiatric labels. This was to improve clarity during the consultation, minimize stigma and increase perinatal women’s engagement with services. Participants noted the significance of this adaptation, stating, “When I read in Krio, I understand better”. Locally recognized idioms for psychological distress, such as “*poil at*” and “*tink too much*”, were integrated to provide a broader understanding of symptoms beyond conventional classifications. Participants highlighted the importance of using relatable terms: “*poil at*” feels less stigmatizing compared to depression, making it easier to talk about psychological distress.

The model also highlights the importance of client-therapist pairing, focusing on shared cultural backgrounds and community acquaintance. MMSGs, who are ethnically and gender-matched to the communities they serve, foster trust and credibility. One participant remarked, “because she understands us, I can speak freely”. Their training in the FBI allows them to build empathic, non-judgmental relationships, enhancing client engagement. MMSGs emphasize confidentiality, with one stating, “We always remind them that what is shared stays between us”. The content adaptation involved integrating culturally relevant case studies and addressing problems during community interactions. MMSGs were trained to respect local practices, including traditional remedies. The intervention addresses common stressors and promotes social connections. Participants emphasized the importance of integrating local remedies: “Mixing traditional practices with new ideas helps us feel supported”. The focus on behavioural activation encourages the rediscovery of enjoyable activities, such as braiding and visiting friends, reinforcing a holistic view of well-being.

The EVM stresses that intervention concepts must align with cultural norms and be understood by clients, promoting positive inter-partner relationships and reducing violence. The PST intervention adopts a bio-psycho-social-spiritual framework, highlighting the interconnectedness of physical symptoms, thoughts, and emotions. One participant noted, “Understanding that my feelings can affect my body helps me seek help”.

Methods were tailored to individual sessions due to clients’ preferences for privacy, ensuring flexibility to accommodate personal commitments. The intervention minimizes written materials and uses pictures, graphics, and drawings (**Additional Files I–III** in **Supplementary Material**) This was intended to make the locally developed and validated screening tool for perinatal psychological distress and the function scale (16), as well as the assignment and activity tracker for perinatal women more user-friendly for women with low literacy and numeracy.. One participant noted, “The pictures help me see what I need to do”. The structured problem-solving approach, reinforced by the manualized materials and consistent supervision, ensures clarity and understanding throughout the therapeutic process.

Contextual factors—such as social support, stigma, and transportation—were considered in designing the intervention. Flexibility in scheduling therapy sessions around significant local events enhances acceptability. A participant remarked, “Having sessions at times that work for us makes attending easier. “The intervention’s adaptability to community dynamics, such as family involvement and culturally appropriate delivery methods, underscores its relevance. Participants emphasized the importance of involving male partners in discussions, stating,” When my husband knows, he can support me better.” Metaphors which are sayings that are familiar to the community were integrated, such as “*Ol kondo de dreg in belleh na gron, yu kno kno d wan wae in belleh de at”* meaning ‘some challenges in life might be invisible’. Participants appreciated this approach, with one saying, “Stories from our culture make the messages clearer”. The use of expressions like” “*fambul tik kin ben, but enoba brok*”, meaning ‘families endure challenges over time but consistently recover’, this resonates deeply with the community members. The goals of treatment were designed to reflect and reinforce positive cultural values, focusing on the client’s perspective and promoting optimal symptom reduction and social functioning. The EVM emphasizes a comprehensive, culturally sensitive framework for effective intervention delivery. Participants stressed the importance of clarifying treatment expectations, with one stating, “We need to know what to expect; it helps reduce our fears”. Education about stigma and misconceptions surrounding mental health is crucial for engagement.

#### Phase 4: production

**Figure 1:**
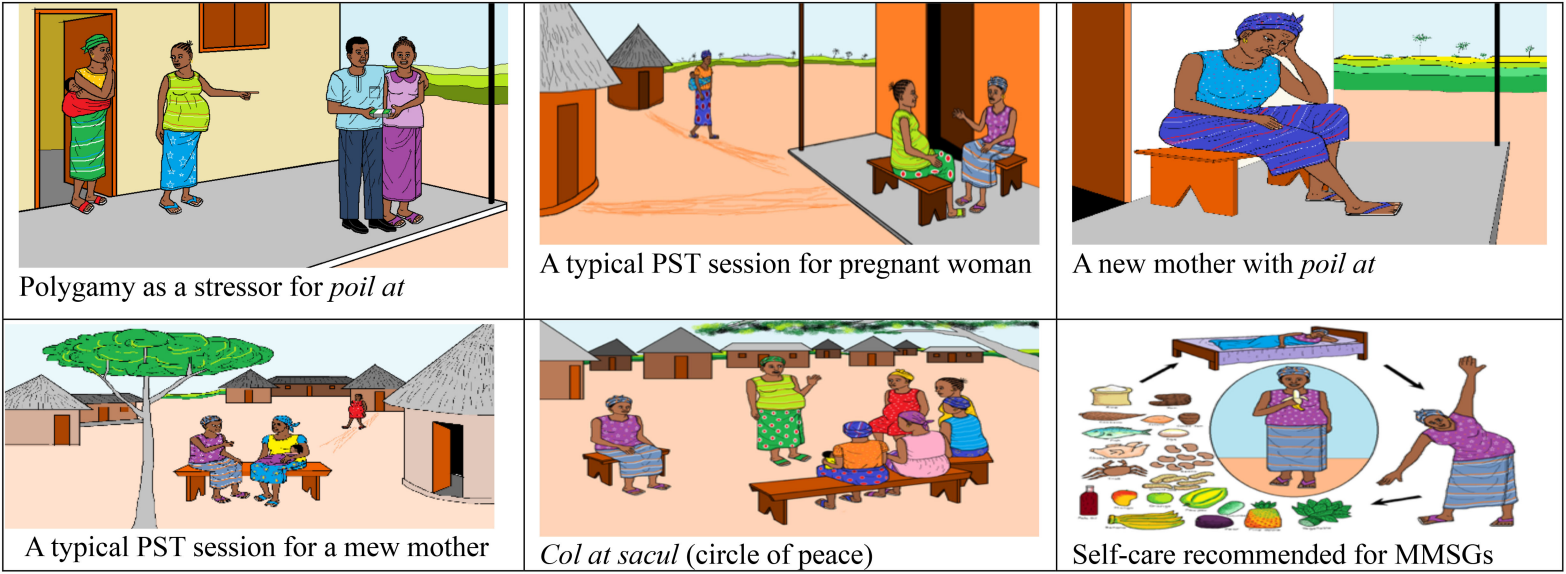
Model illustrations included in the adapted manual.

After imputing the contributions from the MMSGs, primary healthcare workers, and the perinatal women, we produced a fifteen-chapter manual intended to guide the piloting of the culturally adapted intervention. To effectively manage the delivery of the intervention by MMSGs with low literacy levels, we proposed several strategies: utilizing visual aids such as pictures and diagrams to convey key concepts (see **Figure 1** for a model illustration), emphasizing oral communication and storytelling as primary methods for sharing information. Training lay MMSGs to facilitate discussions and document key insights, and implementing simple feedback mechanisms to gauge understanding without requiring written responses, thereby making the intervention more accessible and effective. Additional File 1 and 2 provide screening tools (48) for psychological distress and functional capacity, translated in Krio and presented in graphic form, while Additional File 3 depicts the culturally adapted FBI, including the behavioral activation components in graphic design, and a weekly calendar using the sun as a reference frame for the time of the day, to remind perinatal women of specific activities to incorporate into their weekly plans following the PST sessions.

#### Phase 5 and 6: topical expert and integration

The topical experts recommended adjustments to ensure the vignettes and metaphors reflected the formative, qualitative data from Sierra Leone, while maintaining the core structure of the intervention. The committee emphasized preserving the fidelity of in-person counselling procedures and maintaining the PST framework in each session.

The integration phase involved the research team meeting to iteratively revise the manual based on feedback from the topical experts. This collaborative effort led to the development of version 4.0, the final version. This version intends to train MMSGs in a randomized controlled, pilot study to test the feasibility, acceptability and preliminary effectiveness of the culturally adapted intervention (see **Table 5**).

**Table 5:** Summary of changes in the Friendship Bench Manual.

Chapter	Chapter FB Manual	Revision
N/A	Cover page	• Changed the illustration from the face of an old woman to that of an old woman sitting under a tree on a bench talking to a new mother, with a pregnant woman walking towards them, with thatch houses distributed around as in a village setting • Lay health worker changed to mother-to-mother support groups to reflect the lay women implementing the intervention
1	Introduction	Rewrote the introduction as a background to fit the purpose of the program in Sierra Leone. In addition to the aim and process covered in the Zimbabwean manual, we also covered the structure of the manual, who can use it, briefly on training and supervision.
2	Historical background of the friendship bench and justification	Removed
3	Psychoeducation	• We removed “kufungisisa” (Zimbabwean for depression and anxiety) and replaced it with the symptoms from the rapid ethnographic study such as heart related idioms of distress for emotion e.g., “*poil at*” “*at nor swit*” and mind relation idioms such as “*no peace of mind*” or “*mind not steady*” to denote psychological distress • Removed information about tablets since we used paper-based copies.
4	Common mental disorders	Removed and replaced with idioms of distress, signs, symptoms and explanatory model from the rapid ethnographic study such as *“poil at”*, “*thinking too much*” and “*mind not at peace”.*
5	Friendship bench intervention	Merged with Chapter 9
6	Screening tools for SSQ and PHQ-9	Replaced screening CPMD by SSQ and PHQ-9 in Zimbabwe by two tools developed in the parent study, the Sierra Leone Perinatal Psychological Distress Scale (SLPPDS) and the Function Scale (48).
7	Counselling skills	Retained
8	Friendship card	Retained but instead of the smart action plan, goals, problem and next appointment, ours also had a calendar that shows the seven days of the week and the rising of the sun, when it’s over head and sun set in pictures to guide them, when to implement the agreed action points, because of their low literacy. A diagram that depicts behavioural activation was also on the card to encourage the client to re-activate behaviours that they had found positive in the past
9	Problem solving therapy	Retained but modified instead of seven steps to four steps
10	Strong emotional reactions	Replaced this with stress as a topic, types, presentations and complications if remain untreated. Two weighing scales diagrams was used as an illustration to show when the perinatal woman is balance and unbalanced with regards emotions
11	Suicide assessment	Retained but as an appendix
12	Supervision	Retained
13	Home visit	Retained
14	Circle	Retained but replaced “*kubatana tose circle*” with “*col at sacul*” (calmness/peace circle)
15	Self-care	Retained
16	Proverbs	Replaced with Sierra Leonean proverbs and metaphors e.g., ‘*if yu tek tem kill anch, yu go see in gut*’ meaning keeping one’s cool; ‘*good wod pull kola*’ meaning to be focus on the positive.
17	Training overview	Changed the training format from 9 days to 2 days and rewrite the training programs that reflect the new manual and the context, with use of images and graphics and less texts to reflect the low literacy of the MMSGs
18	Shona training material	Removed
19	Others	Generated case studies and vignettes from the key informant interviews in the qualitative study to replace the ones in the manual, tailored to the Sierra Leone context and use names common in Sierra Leone such as Hawa, Adama and Sia.

CPMD, Common perinatal mental disorder.

FBI, Friendship bench intervention.

MMSG, Mother to mother support group.

N/A, Not applicable.

PPD, Perinatal psychological distress.

RES, Rapid ethnographic study.

SSQ, Shona symptoms questionnaire.

PHQ 9, Patient health questionnaire.

#### Phase 7 and 8: training and testing

These phases focused on training and testing the culturally adapted intervention. We conducted a two-day training for the MMSGs that piloted the culturally adapted intervention at intervention and control sites. Subsequently, a randomized controlled pilot study was conducted to assess the intervention’s feasibility, acceptability and preliminary effectiveness among newly screened pregnant women and new mothers, published in a subsequent manuscript. The findings from this mixed-method pilot study will inform the final version of the culturally adapted FBI manual that would be used for a well-powered randomized controlled Trial.

## Discussion

This study systematically adapted an evidence-based mental health intervention originally designed for common mental disorders among adults in Zimbabwe to address the needs of perinatal women in Sierra Leone (21, 26). Using community-based participatory research methods, guided by the ADAPT-ITT framework and Bernal’s EVM (49), we collaborated with community members, primary healthcare workers, and perinatal women to achieve the cultural adaptation. This approach ensured that the life-world of perinatal women were integral to the cultural adaptation, enabling the intervention to effectively address their specific needs and priorities. A key feature of the adapted intervention is its lay health worker delivery model, where trained MMSGs, who do not have formal mental health training, will be implementing the intervention. Research has shown that lay health worker delivery models can be effective for mental health interventions in low-resource settings (25, 50, 51).

Our findings underscore the importance of culturally sensitive adaptations of evidence-based interventions, which resonate with the specific needs of the target population and enhance engagement and effectiveness (52). Previous adaptations of the FBI have demonstrated it feasibility and effectiveness of culturally tailored interventions in various settings (27, 37, 46, 53). For instance, the FBI was successfully adapted for use in Zimbabwe, where community health workers delivered PST to address common mental disorders, significantly improving mental health outcomes (36, 54, 55). This study exemplifies how community involvement and local context can enhance the efficacy of mental health interventions.

Previous studies have shown that culturally adapted interventions conducted by lay health workers in sub-Saharan African countries significantly enhanced the effectiveness of health programs. Noteworthy examples include the IPT in South Africa (44, 45), IPT in Uganda (40–43), PST in Zimbabwe (25, 36), and CBT in Zambia (39). Although some of these studies employed randomized control trial methodologies (25, 40–43), others utilized quasi-experimental designs (56), non-randomized approaches (36, 44), cohort prospective studies (39), and randomized control pilot studies (45). It is important to note that the application of a cultural adaptation framework was largely absent in most of these studies, with cultural adaptation often encompassing only a limited scope. The elements of cultural adaptation included the reduction of training and intervention delivery times, the use of local languages, the integration of cultural and religious components, and the involvement of local lay health workers (57). All of these adaptations have demonstrated effectiveness and acceptability.

Our adaptation process, involving community feedback from the MMSGs and perinatal women, ensured that the intervention was grounded in scientific evidence while also reflecting the lived experiences of the pregnant women and new mothers (57). This participatory approach is consistent with the principles of community-based participatory research, emphasizing collaboration and co-learning between researchers and community members (58). The cultural adaptation highlighted the interplay between societal norms, traditional beliefs, and the psychological distress experienced by perinatal women, which aligns with findings from other contexts where culturally tailored interventions have been shown to increase engagement with services, reduce stigma, and improved the outcomes of the target population (59).

### Limitations and future directions

While our adaptation process was robust, it is essential to acknowledge some limitations. Firstly, relying on the MMSGs for perinatal mental health support can lead to inconsistencies in the quality of care provided. While these community members can offer valuable assistance, they often lack the professional training and expertise necessary for addressing complex mental health issues. This variability may affect the effectiveness of the intervention, particularly for more severe cases that require specialized care from trained professionals. Furthermore, the long-term sustainability of such interventions is a significant concern. Continuous funding, training, and supervision of the MMSGs are crucial to maintain the quality and impact of the program.

Additionally, evaluating the effectiveness of the intervention poses difficulties, particularly in capturing long-term outcomes and ensuring that the goals are met across diverse settings. Addressing these challenges is essential to enhance the overall impact and success of the culturally adapted FBI intervention for perinatal psychological distress in Sierra Leone.

### Implications for maternal mental health

The culturally adapted FBI for perinatal mental disorders in Sierra Leone offers significant benefits by aligning with local cultural beliefs, enhancing its relevance and acceptance among perinatal women. By utilizing community resources and the MMSGs, the intervention increases accessibility to mental health support, especially in rural or underserved areas where professional services are limited. This approach helps overcome logistical and financial barriers that perinatal women often face, ensuring they receive the support they need during a vulnerable period.

Furthermore, the intervention fosters vital social support networks, which can alleviate feelings of isolation and anxiety among perinatal women. By normalizing discussions about mental health within the community, the FBI helps reduce stigma, encouraging more women to seek help. The success of this culturally sensitive model in Sierra Leone could serve as a blueprint for similar initiatives in other regions, ultimately improving maternal and infant health outcomes and contributing to a more supportive and resilient community.

## Conclusion

In conclusion, the cultural adaptation of the FBI for perinatal psychological distress in Sierra Leone represents a meaningful advancement in addressing the mental health needs of women during the perinatal period. This study provides valuable insights for developing effective mental health interventions in LMICs by using a culturally informed, community-driven approach. The successful adaptation of the FBI provides a model for future interventions. It highlights the necessity of tailoring mental health services to the unique cultural contexts of the populations they serve. The next step will be to assess the feasibility, acceptability and preliminary effectiveness of the intervention and its delivery in perinatal care settings.

## Data Availability

The original contributions presented in the study are included in the article/**Supplementary Material**. Further inquiries can be directed to the corresponding author/s.
