# Supplementary material for: The cultural adaptation of the Friendship Bench Intervention to address perinatal psychological distress in Sierra Leone: an application of the ADAPT-ITT framework and the Ecological Validity Model
Source: Front Psychiatry. 2025 Feb 19;16:1441936. doi: 10.3389/fpsyt.2025.1441936 (PMC11880789; doi:10.3389/fpsyt.2025.1441936)
Supplement: Supplementary file 1 [file DataSheet1.docx]

**SUPPLEMENTARY TABLES**

**Additional files**

**Additional file I**

1. **Sierra Leone Perinatal Psychological Distress Scale (SLPPDS)**

Introduction of scale

Insay di las 2 wik dɛm, ɔmɔs tɛm dɛn prɔblɛm dɛm ya dɔn mɔna yu? (yuz “✓” fɔ sho yu ansa)

Over the past 2 weeks, how often have you been bothered by any of the following problems? (Use “✓” to indicate your answer)

| Tin dɛm fɔ du  Activities | No, not at all  Nɔ, Nɔ apin at ɔl  *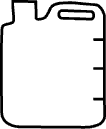* | No, not much  Nɔ, nɔ apin bɛtɛ 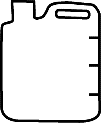 | Yes, sometimes  Yɛs, sɔm tɛm dɛm 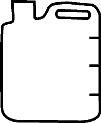 | Yes, quite a lot  Yɛs, plɛnti tɛm 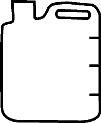 |
| --- | --- | --- | --- | --- |
| Yu dɔn fil lɛk yu nɔ gladi? / Have you felt sad?  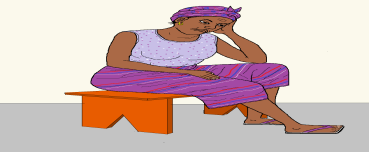 | 0 | 1 | 2 | 3 |
| Yu dɔn fil lɛk I mɔna fɔ yu fɔ slip na net? / Have you been so unhappy that you have had difficulty sleeping?  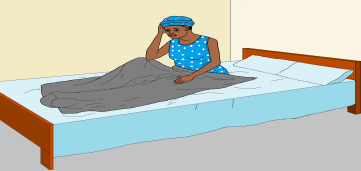 | 0 | 1 | 2 | 3 |
| Smɔl tin dɔn mek yu cray? /Have you cried easily?  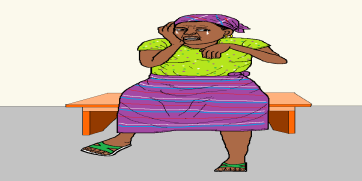 | 0 | 1 | 2 | 3 |
| Yu dɔn tray fɔ kip to yusɛf? / Have you tried to be alone?    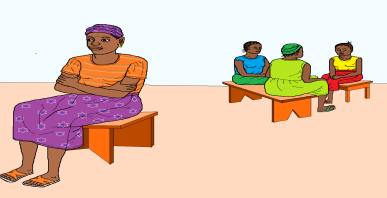 | 0 | 1 | 2 | 3 |
| Yu dɔn vɛx kwik kwik wan? / Have you been getting angry easily?  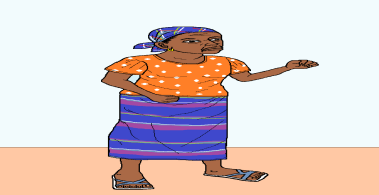 | 0 | 1 | 2 | 3 |
| Yu dɔn tink fɔ du bad to yu man?  Have you thought o harming your husband?  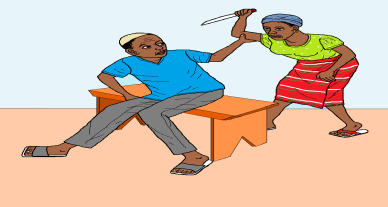 | 0 | 1 | 2 | 3 |
| Yu dɔn fil diskɔrej, lɛk yu at nɔ swit? / Have you been feeling discourage?  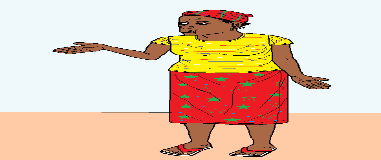 | 0 | 1 | 2 | 3 |
| Yu dɔn de tink tink bɔku?/ Have you been thinking too much?  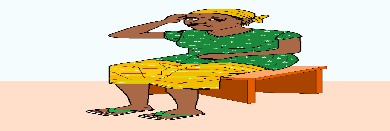 | 0 | 1 | 2 | 3 |
| Yu dɔn tɔk tɔk pan yusɛf?27/ Have you been talking to yourself?  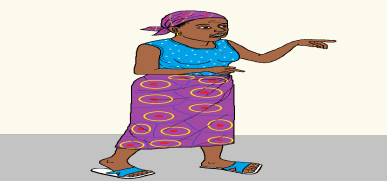 | 0 | 1 | 2 | 3 |
| Yu dɔn fil shem? / Have you felt ashamed?  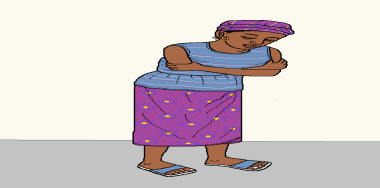 | 0 | 1 | 2 | 3 |

**Additional file II**

1. **Function Scale**

*Fɔchɛk aw di pɔsin de du*

*Checking how the person is doing*

*A go rid wan list we gɛt tin dɛm we bɛlɛ uman/kombra dɛm kin du. Fɔɛni wan pan dɛn tin ya,*

*I will read a list bearing thing done by pregnant and young mothers. For each of these*

*a go askyu aw I at fɔyu fɔ du pas yu kɔmpin bɛlɛuman/kombra dɛm.*

| **Tin dɛmfɔ du**  **Activities** | **Aw I dɔn at fɔ du dɛn tin ya**  **How difficult it is for you in doing these activities** | | | | |
| --- | --- | --- | --- | --- | --- |
|  | ****  I nɔ at I at smɔl I atgbeŋ I at gbeŋgbeŋ Nɔ kin ebul du amsɛf | | | | |
| Os wok dɛm (fɔbruk, swip, Fɔkuk ,klinos)  Household chores (laundry, sweeping, cooking cleaning)  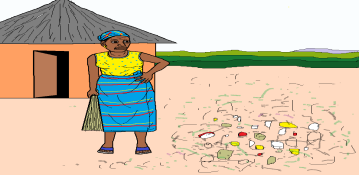 | 0 | 1 | 2 | 3 | 4 |
| Tin dɛm we de bring mɔni (fam wok, fɔ bay ɛn sɛl ɔ fɔ wok)  Income generating (farming, petty trading or work)  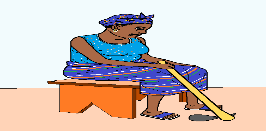 | 0 | 1 | 2 | 3 | 4 |
| Fɔ du di tin dɛm we yu lɛk(wach fim, ɛksasayz,go fɛnpadi)  To do things you like (watch movies, exercise, visit friends)  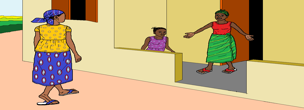 | 0 | 1 | 2 | 3 | 4 |
|  | 0 | 1 | 2 | 3 | 4 |
| Fɔ go na klinic fɔ chɛkɔp  Going to the clinic for check up  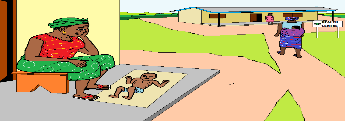 | 0 | 1 | 2 | 3 | 4 |
| Fɔtek pat pan tin dɛm we de apin na yu kɔmyuniti  Taking part in community activities  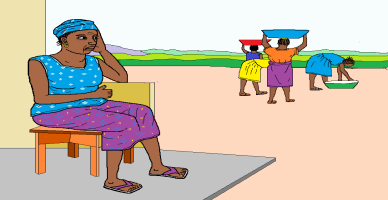 | 0 | 1 | 2 | 3 | 4 |

**Additional file III**

1. **Handout for pregnant women and new mothers to serve as reminders and trackers**

The following steps are to be followed by you as a pregnant woman or a new mother.

- **Managing Problems**
- **Get Going Keep Doing**
- **Weekly Calendar**

The Calendar can be used to record when you will complete various activities (e.g., Activities from the Action Plans of Managing Problems or Get Going, Keep Doing).

**Steps to Managing Problems**

|  | **Problem solving therapy steps** | **Sierra Leone Friendship bench** | **STEP** |
| --- | --- | --- | --- |
| 1 | List problems | “*Tell am ol yu belleh word*”  (list all problems) | 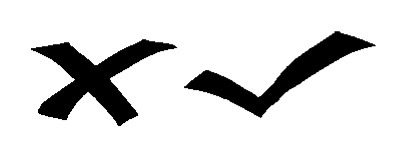 |
| 2 | Set realistic goals | ‘*Heng hed’*  (put heads together/brainstorm) | 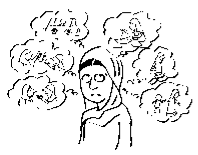 |
| 3 | Generate solutions |  |  |
| 4 | Evaluate and compare solutions | ‘*Fen way for make tin go bifo*”  (Identify a solution for implementation) | 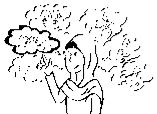 |
| 5 | Select feasible solution |  |  |
| 6 | Implement solution |  |  |
| 7 | Evaluate outcome | “*Chek if bette de*”  (evaluate outcome) | 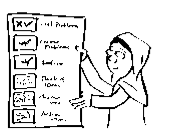 |

**Get going, keep going**


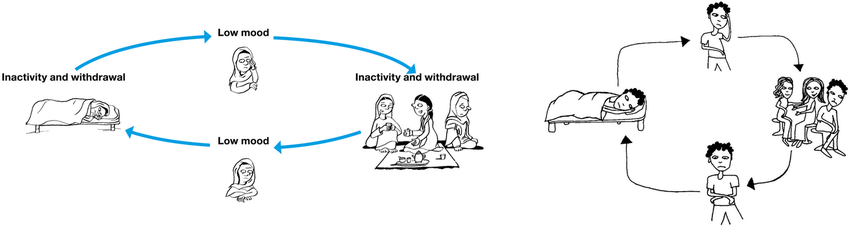


**Weekly calendar**

| **Time** | Monday | Tuesday | Wednesday | Thursday | Friday | Saturday | Sunday |
| --- | --- | --- | --- | --- | --- | --- | --- |
| 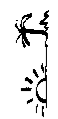  **Morning** |  |  |  |  |  |  |  |
| 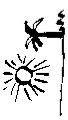  **Afternoon** |  |  |  |  |  |  |  |
| 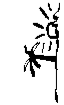  **Evening** |  |  |  |  |  |  |  |

**Additional file IV**

1. Details of the cultural and contextual adaptation plan of the problem-solving therapy using Bernal’s framework

| **Adaptation principle** | **Operationalization** | **Rationale** |
| --- | --- | --- |
| **Language** | | |
| **Translation into local language** | Training manuals, helpers guide and client’s materials were translated into the local language, Krio. | To match the language spoken by clients and the MMSGs to enhance understanding of the intervention’s concepts, methods and goals. |
| **Use of local idioms of perinatal psychological distress** | Identification of frequently used local idioms of psychological distress from the formative study: *poil at, heng at, stres, thinking too much, at nor swit, discourage, and frustrate.* | Local perinatal psychological distress contained symptoms in addition to those described in DSM-IV & ICD 10. |
| **Technical terms replaced by colloquial expressions** | All technical terms were translated into colloquial expressions and  replace the term ‘depression’ with vernacular, culturally congruent terms such as ‘*poil at’, ‘at nor swit’*; and ‘anxiety ‘, with ‘*stres’* or ‘*worry*’ and avoidance of psychiatric labels as was in the original manual | Using literal translations or translations which are not culturally appropriate is one of the major barriers in therapy. Depression not well understood as a term. *Stres, thinking too much or frustrate* are more understandable in the local context. And also minimize stigma and increase engagement with services. |
| **Therapist** | | |
| **Therapist –patient matching** | MMSGs ethnically and gender matched, and from the same local community, speaking the local language. | Local credibility and acceptability, fluency in local dialect, shared lived experience, norms and events impacting community. In addition, familiarity with the local idioms of distress. Respect for local gender values, to promote self-disclosure, |
| **Cultural competence** | The MMSGs will be trained in cultural competence and they have considerable experience working with pregnant women, encouraging them to attend their antenatal care and self-hygiene. They also encourage new mothers to practice exclusive breastfeeding and attend postnatal care. | To engage with the pregnant women and new mothers and make treatment culturally relevant |
| **Therapist-patient relationship** | The MMSGs works as a guide adopting a more directive approach. MMSG should avoid taking the lead, their role is more of a facilitative role | African concept of "mentor" is different from western concept of Socratic dialogue leading to change - clients unable to identify solutions and looked to the counsellor for direction |
|  | Emphasis made on active listening, developing an empathic, and non-judgmental relationship with the client. | To ensure client engagement in the treatment process |
|  | MMSGs trained in setting boundaries within the therapeutic relationship with the client. | To structure the informal nature of the home setting |
|  | Participants will be addressed and greeted in a traditional manner and at the beginning of each session they will be reminded about confidentiality & privacy. | To make the engagement process more effective. |
| **Use of non-mental health workers** | Use of MMSGs who are already serving in a voluntary role at community level, respected and trusted in the community as they are hand-picked and recommended by the community. | To reduce stigma and preserve patient’s privacy (especially during home visits) from inquisitive neighbours/family members. Also, to make best use of already available and/or low-cost resources, as the existing health workforce is already over stretched |
| **Metaphors** | | |
|  | Use of drawings provided to each mother to monitor homework. Using culturally appropriate illustrations, for example, characters depicting the problems to be addressed. | It will facilitate work with non-literate women |
| **Use of stories, local examples** | Examples from religious texts (the life of the Prophet Muhammad in the Quran or Jesus Christ in the Bible will be used to clarify issues) | Perinatal women could understand new ideas when described using familiar stories/figures and enhance acceptability of treatment |
|  | Metaphors were integrated in the training curriculum and the intervention manual (e.g., “*fambul tik kin ben, bt enoba broke*”), meaning family focus to encourage family engagement; *Ol kondo de dreg in belleh na gron, yu kno kno d wan wae in belleh de at (Krio)….* meaning, we all experience stress at some point in our lives, you can only understand what people are going through sometimes when you talk to them). The use of common sayings was important for this intervention because the use of short sayings with powerful meaning is culturally salient across Sierra Leone. The use of drawings, symbols, illustrations depicting the problems they will be working on was also found very useful and culturally appropriate | To increase cultural relevance |
| **Content** | | |
| **Addressing stressors** | Identifying stressors triggering psychological distress and working on breaking social isolation, and building skills to handle stressors more effectively. | To overcome psychological distress by providing a clear context to understanding the symptoms; Mobilize strategies, e.g., break social isolation, problem solving, identify local supports |
| **Incorporation of local practices into treatment** | Tolerate local remedies and practices, in addition to the ‘core’ treatment, for example, attending religious practices and ceremonies. The MMSGs should be respectful towards patient’s practices which are culturally acceptable, for example use of traditional medicines, attending faith healers/spiritual healers | To contextualize the treatment to address issues that are relevant to the cultural group |
| **Additional therapy modules to address cultural factors** | Culturally specific sections of content added as therapy modules. These include: 1) creating healthy relationships; 2) spirituality; 3) Respect for the partner and elders that reflect the local norms, and 4) Sierra Leonean female identity | To contextualize the therapy manual to address issues relevant to Sierra Leonean women |
|  | Greater focus also on teaching perinatal women in identifying things they use to enjoy doing before that they found no longer interesting (behavioural activation) such as visiting friends, listening to music, and watching movies. | To help them reactivate these activities, which will help them to increase their quality of life |
| **Concepts** | | |
| **Somatic** | The bio-psycho-social-spiritual model will be used. Similarly, somatic symptoms will be included to highlight the importance of the physical symptoms and their association with thoughts and mood. | To preserve congruence with cultural beliefs and physical/somatic belief models of illness causation |
| **Social** | “Deconstructing” poverty as a series of role transitions (either loss of finances/resources or failure to bring on a desired improvement | To avoid over attribution of depression to poverty, promote more discussion of interpersonal and social roles that have been impacted by poverty, and mobilize support systems to assist in its improvement |
|  | Loneliness and social isolation were identified as problem, encourage client to establish social contacts | People were socialized and participated in community activities on a daily basis |
|  | Engagement sessions in problem solving therapy to address basic needs | To identify barriers to care, and increase likelihood of participation in treatment to make treatment culturally relevant to and effective for depressed mothers and expectant mothers on low incomes for whom multiple stressful social problems and depression are closely linked |
|  | Providing the "sick role," e.g., assigning of ‘*poil at’ and ‘stres’* as medical conditions that can be treated; in the meantime, patient’s and family’s expectations for optimal functioning need to be reduced temporarily until symptom remission. Patient is encouraged to ask family and community for help and support | To reduce guilt and burden due to symptoms and functional impairment while mobilizing interpersonal resources |
| **Religious** | The MMSGs need to understand the cause-and-effect relationship in th local culture, which might be based on social, cultural or religious causes i.e., causes of illness might be based on a combination of these factors. | To overcome barriers to recruitment and attendance for the subjects |
| **Goals** | | |
| **Client derived goal** | Focus on the client rather than the psychological distress and have an a priori plan of achieving optimal symptom reduction and improvement in social function. | Focus should be to address the problems in the social world of the client and if possible, help her mobilize family support |
| **Clarifying goals** | Emphasis on non-material goals, e.g., improving psychological distress and social functioning, rather than provision of material goods or services | Clarification of treatment expectations; community accustomed to receiving financial and health assistance from NGOs |
| **Extending goals beyond psychological distress** | Emphasis on educating patient, while respecting their understanding and explanation of their problems, especially addressing stigma and misperceptions | Patients generally had no or minimum knowledge of illness and its treatment, especially psychotherapy |
| **Methods** | | |
| **Adaptation in techniques used to deliver treatment** | Less use of written material and limiting homework to simple suggestions rather than writing tasks | To overcome the challenges associated with limited literacy and numeracy |
|  | Structured problem solving with the help of illustrations: e.g., in each session mothers will be shown pictures to identify the triggers. The pictures will be used to facilitate the therapy sessions. | MMSGs are able to understand and employ these techniques and they are easily understandable by the pregnant women and new mothers. |
|  | Each session employs the three-step approach that is repeated throughout the programme | Simplification of the methods by dividing it into simple steps is necessary so that MMSGs are able to understand and employ these techniques. |
|  | Explicit treatment protocols with supporting materials; weekly supervision of all the MMSGs; manualized materials used with all clients, who are given choices and set the pace of the intervention. | Fidelity achieved through standardization without sacrificing the perinatal women’s autonomy and choice |
|  | Avoidance of culturally inappropriate styles of communication. | To assist the building of problem-solving skills while respecting local codes of conduct |
|  | Use of handouts: with simplified pictures that explains the psychological intervention for pregnant women and new mothers | To make treatment understandable |
| **Context** | | |
| **Increase accessibility** | Flexibility in scheduling and conducting sessions, for example, so patients could attend important village-wide events (e.g., funerals) and provisions made to allow for interruptions during sessions | Sensitivity to local community customs to maximize acceptability of the treatment and improve buy-in from wider community and respect the client’s autonomy and choice |
|  | Delivering interventions in a culturally acceptable, close to the community health centre -- "one-stop-shop" or “home visit”. The former will allow them to seek permission from their partners and simultaneously attend their antenatal Care (ANC) or postnatal care (PNC) before or after the sessions | To increase access to care, referral from primary care, and reduce client burden |
| **Ensure feasibility** | Time keeping through the time kids go to school, women go to market or their husband go to the farm, rather than through watches | Participants view time of day according to these tasks for the most part. |
|  | MMSG may visit or send coded verbal messages to remind clients of the time, day and date of sessions | To ensure adherence |
| **Ensure acceptability** | Treatment delivered on individual basis, as individual format preferred from our formative study due to concerns about confidentiality | To facilitate disclosure which may be hampered in group sessions where gossiping is common in these communities, and might expose them to shame |
|  | Disclosure to community about the program through stakeholder’s engagement. | To avoid suspicion, promote buy-in and ensure the community own the program for its sustainability |
|  | The adaptations were done specifically to allow the inclusion and retention of low-income mothers; extensive adaptations to allow for harsh circumstances and stressors as well as single-parent status, acknowledgement of genuine threats (e.g., abusive partner, current separation from family). | Contextual stressors were seen as one of the major contributors to depression |
|  | Inclusion of family members in treatment if preferred by perinatal women | Acknowledges the central role of the family in the treatment process |
|  | In Sierra Leone, the male partner’s and husband’s approval is key; we will brief them about the therapy, accord them their respect and trust during the home visit if the woman is ok with that. | Acknowledgement of the traditions and values allowed the therapy teams entry into these families and increased the possibility of follow up |
